# Association between urine pH on ICU day 1 and 90-day mortality in sepsis: a retrospective cohort study

**DOI:** 10.62838/jccm-2026-0042

**Published:** 2026-10-30

**Authors:** Tomoki Tanaka, Kazunori Fujino, Yasuyuki Tsujita, Yuka Segoe, Yugo Matsumoto, Mitsuhiro Fujino, Hidemitsu Miyatake, Naoto Mizumura, Junji Shimizu, Takuma Kishimoto, Naoto Shiomi

**Affiliations:** Department of Critical and Intensive Care Medicine, Shiga University of Medical Science, Otsu, Japan; Shiga University of Medical Science Hospital, Emergency and Intensive Care Unit, Otsu, Japan

**Keywords:** Sepsis, Urine pH, dipstick urinalysis, intensive care unit, risk stratification

## Abstract

**Introduction:**

Urine pH is an easily obtainable bedside indicator of urinary acidification; however, its prognostic value in sepsis remains unclear.

**Aim of the study:**

We investigated the association between urine pH measured on intensive care unit (ICU) Day 1 and 90-day mortality in patients with sepsis.

**Material and methods:**

This single-center retrospective cohort study was conducted at Shiga University of Medical Science Hospital between August 2011 and April 2023. Adult ICU patients with sepsis who had urine pH measured on ICU Day 1 were included. Urine pH was assessed using dipstick colorimetry, and the earliest value within 12 h of ICU admission was analyzed. The primary outcome was 90-day all-cause mortality. Associations were evaluated using multivariable Cox proportional hazards models.

**Results:**

Among 468 patients (median age 72 years; 61.8% male), the 90-day mortality rate was 25.6%. Spline analysis showed no evidence of nonlinearity (P=0.331). Each 0.5-unit increase in Day-1 urine pH was associated with lower 90-day mortality (adjusted hazard ratio [HR] 0.572; 95% confidence interval [CI] 0.422–0.776; P<0.001). Mortality was 30.2% in patients with urine pH ≤6.0 and 11.4% in those with urine pH >6.0; higher urine pH remained associated with lower mortality risk after adjustment (adjusted HR 0.279; 95% CI 0.147–0.529; P<0.001). Urine pH on ICU Days 2 or 3 was not significantly associated with mortality.

**Conclusions:**

Lower urine pH on ICU Day 1 was independently associated with increased 90-day mortality in sepsis, suggesting that urine pH may complement early risk stratification in critically ill patients.

## Introduction

Sepsis is a life-threatening condition characterized by organ dysfunction resulting from a dysregulated host response to infection, and remains one of the leading causes of death in the field of critical care [1]. Sepsis-3 definitions have provided a refined framework for the diagnosis, assessment of organ dysfunction, and severity stratification [2]. Nonetheless, sepsis continues to pose a significant global health burden, with a high number of sepsis-related deaths reported worldwide and substantial mortality rates in clinical settings [3].

Accurate early stratification of prognostic risk upon intensive care unit (ICU) admission is crucial for optimizing treatment intensity and improving communication with patients’ families. In patients with sepsis and critical illness, prognostic assessment commonly relies on established indicators such as organ dysfunction scores, including the Sequential Organ Failure Assessment (SOFA) score and other markers of circulatory and metabolic status [2,4,5,6]. However, there is substantial clinical value in identifying additional indicators that are easily obtainable and cost-effective.

In sepsis, circulatory disturbances and metabolic abnormalities frequently lead to acid–base imbalance. Elevated serum lactate levels and blood gas parameters are associated with poor clinical outcomes [4,5,6]. In response to metabolic acidosis, the kidneys enhance acid excretion, including increased ammonium (NH_4_^+^) elimination; however, this acid–base regulatory response may be impaired in critically ill conditions [7,8]. Consequently, alterations in urinary acidification may reflect both systemic acid–base disturbances and renal tubular dysfunction. Several urinary biomarkers have been investigated as tools for the assessment of kidney injury and risk stratification in critically ill patients [9]. However, many of these biomarkers require specialized assays and are not routinely available in daily clinical practice. In contrast, urine pH can be obtained rapidly and inexpensively as part of routine urinalysis. Urine pH provides a convenient surrogate for assessing urinary acidification, but it does not directly reflect NH_4_^+^ excretion or total acid excretion, limiting its interpretability [7]. Although several studies have investigated the association between urine pH and renal outcomes, such as the progression of acute kidney injury (AKI) in critically ill populations [10,11,12,13], the relationship between urine pH and mortality, specifically in sepsis, as well as the prognostic relevance of temporal changes in urine pH during the acute phase, remains unclear.

The primary objective of this study was to evaluate the association between urine pH on the first day of ICU admission and 90-day mortality in patients with sepsis. As a secondary objective, we aimed to assess the relationship between urine pH and organ dysfunction, as measured by SOFA score and urine output, to explore the clinical significance of urine pH. Additionally, as an exploratory analysis, we examined the association between temporal changes in urine pH and 90-day mortality.

## Methods

### Setting and study design

This single-center retrospective observational study was conducted at Shiga University of Medical Science Hospital (Shiga, Japan) between August 2011 and April 2023. We included patients who were admitted to the ICU during the study period, met the Sepsis-3 criteria for sepsis or septic shock, and had measurable urine pH on the first day of ICU admission. For patients admitted before 2016, Sepsis-3 criteria were applied retrospectively to determine eligibility. Patients aged ≤15 years, those who had undergone cardiovascular surgery, and those receiving maintenance dialysis were excluded.

This study was approved by the Ethics Committee of Shiga University of Medical Science (approval number: R2025-121) and was conducted in accordance with the Declaration of Helsinki. Given the retrospective design of the study, the requirement for informed consent was waived using an opt-out approach.

### Outcomes

The primary outcome was 90-day mortality and its association with urine pH on the first day of ICU admission was evaluated. As secondary outcomes, we assessed the temporal trends in urine output and SOFA scores (both total and individual components) stratified by urine pH levels. Additionally, in an exploratory analysis, we defined transition groups based on urine pH on days 1 and 3 using a cut-off value of 6.0, and evaluated their association with 90-day mortality.

### Data collection and variable definitions

Baseline data of all participants, including age, sex, body mass index (BMI), SOFA score, survival status, and comorbidities, were collected from the electronic medical record system. Regarding the renal component of the SOFA score, a substantial number of patients in this study underwent continuous renal replacement therapy (CRRT), and serum creatinine levels are susceptible to dilutional effects from dialysis and large-volume fluid administration. Therefore, urine output criteria were prioritized over serum creatinine for renal function assessment. Additionally, clinical data such as blood test results and arterial blood gas measures, were collected, and the earliest values obtained after ICU admission were used for analysis.

### Urine pH Measurement

Urine pH was measured using a colorimetric method with an N-multistix SG-L and a Clinitek Status analyzer (Siemens, Tokyo, Japan). Measurements were performed at least twice daily, and the earliest value within 12 h of ICU admission was used for analysis. When multiple values were available, the result closest to the time of ICU admission was selected.

Urine samples were collected from the indwelling urinary catheters and immediately tested according to the manufacturer's instructions. Trained staff interpreted the results using test strips with a pH scale ranging from 5.0 to 8.5 in 0.5-unit increments.

### Definition of Study Groups

Patients were categorized into two groups based on urine pH measured on the first day of ICU admission: the Low urine pH group (pH ≤ 6.0) and the High urine pH group (pH > 6.0). The cutoff value of 6.0 corresponded to the median urine pH of the study population and was used to classify patients into relatively lower and higher urine pH groups. Additionally, using the same cutoff value (6.0), patients were further classified into four groups based on the combination of urine pH values on Days 1 and 3: Low–Low, Low–High, High–Low, and High–High (in order of Day 1 to Day 3). Comparative analyses were conducted between these groups to explore associations with clinical outcomes.

## Statistical Analysis

### Primary Analysis

We evaluated the association between urine pH on the first day of ICU admission and 90-day mortality. Urine pH was treated as a continuous variable, and a Cox proportional hazards model incorporating a restricted cubic spline (4 degrees of freedom) was used to assess the dose–response relationship and potential nonlinearity, with a reference value set at pH 6.5. Covariates included in the model were age, sex, BMI, SOFA score (excluding the central nervous system [CNS] component), serum lactate level, and the presence of septic shock. The CNS component was excluded because the neurological SOFA subscore is based on the Glasgow Coma Scale (GCS), which may not accurately reflect underlying neurological dysfunction in critically ill patients owing to the effects of sedation, analgesia, and mechanical ventilation. Previous studies have highlighted these limitations of GCS assessment and reported that SOFA scores excluding the GCS component retain prognostic performance in patients with sepsis [14]. In our ICU, many mechanically ventilated patients had already received sedatives and analgesics before ICU admission, and the recorded GCS frequently reflected treatment-related depression of consciousness rather than true neurological impairment. Therefore, to minimize potential misclassification of baseline illness severity, we used the SOFA score excluding the CNS component in the primary analyses.

Nonlinearity was evaluated by comparing the spline model and a linear model using a likelihood ratio test. Because no significant nonlinearity was detected, we proceeded with a multivariate Cox model, treating urine pH as a linear continuous variable.

### Secondary Analyses

Patients were stratified into two groups based on Day 1 urine pH (≤6.0 vs. >6.0), and 90-day survival was compared using Kaplan–Meier analysis with the log-rank test. Furthermore, a Cox proportional hazards model, adjusted for the same covariates used in the primary analysis, was used to evaluate the association with mortality. Changes in SOFA scores and urine output over time were also compared between the two groups.

### Exploratory Analyses

To assess the time-dependent association between urine pH and clinical outcomes, we constructed separate Cox proportional hazards models using urine pH values from Days 1 to 3 as the primary explanatory variables for each day. Analyses for Day 2 and Day 3 were conducted as landmark analyses, including only patients who survived and had their urine pH measured on the corresponding day.

Furthermore, to explore the effect of changes in urine pH over time, we categorized patients into four groups based on combinations of urine pH values on days 1 and 3: Low–Low, Low–High, High–Low, and High–High (with a cutoff of pH 6.0). Within the Day 1 strata, we compared High–High vs. High–Low and Low–Low vs. Low–High. Kaplan–Meier survival curves and the log-rank test were used to evaluate differences in survival among groups. Cox proportional hazards models adjusted for the same covariates used in the primary analysis were applied using the High–High and Low–Low groups as reference categories.

Continuous variables were presented as medians with interquartile ranges (IQRs) and categorical variables as counts with percentages. The Mann–Whitney U test was used for comparisons of continuous variables and Fisher's exact test for categorical variables. Survival curves were generated using the Kaplan–Meier method, and group differences were assessed using the log-rank test. Missing values were not imputed, and analyses were performed using available data (complete-case analysis). All statistical tests were two-tailed, and a P-value <0.05 was considered statistically significant.

All statistical analyses were performed using SPSS version 22 (SPSS Inc., Chicago, IL, USA).

## Results

### Patient Flow and Baseline Characteristics

In total, 10,465 patients were admitted to the ICU during the study period. After excluding 5,513 patients who underwent cardiovascular surgery, 366 aged < 15 years, and 3,952 without sepsis, 634 patients with sepsis were identified (Figure 1). Among them, 40 patients receiving maintenance dialysis and 126 patients without urine pH measurements on Day 1 of ICU admission were excluded (Supplemental Table 1), resulting in a final cohort of 468 patients.

**Fig. 1.**
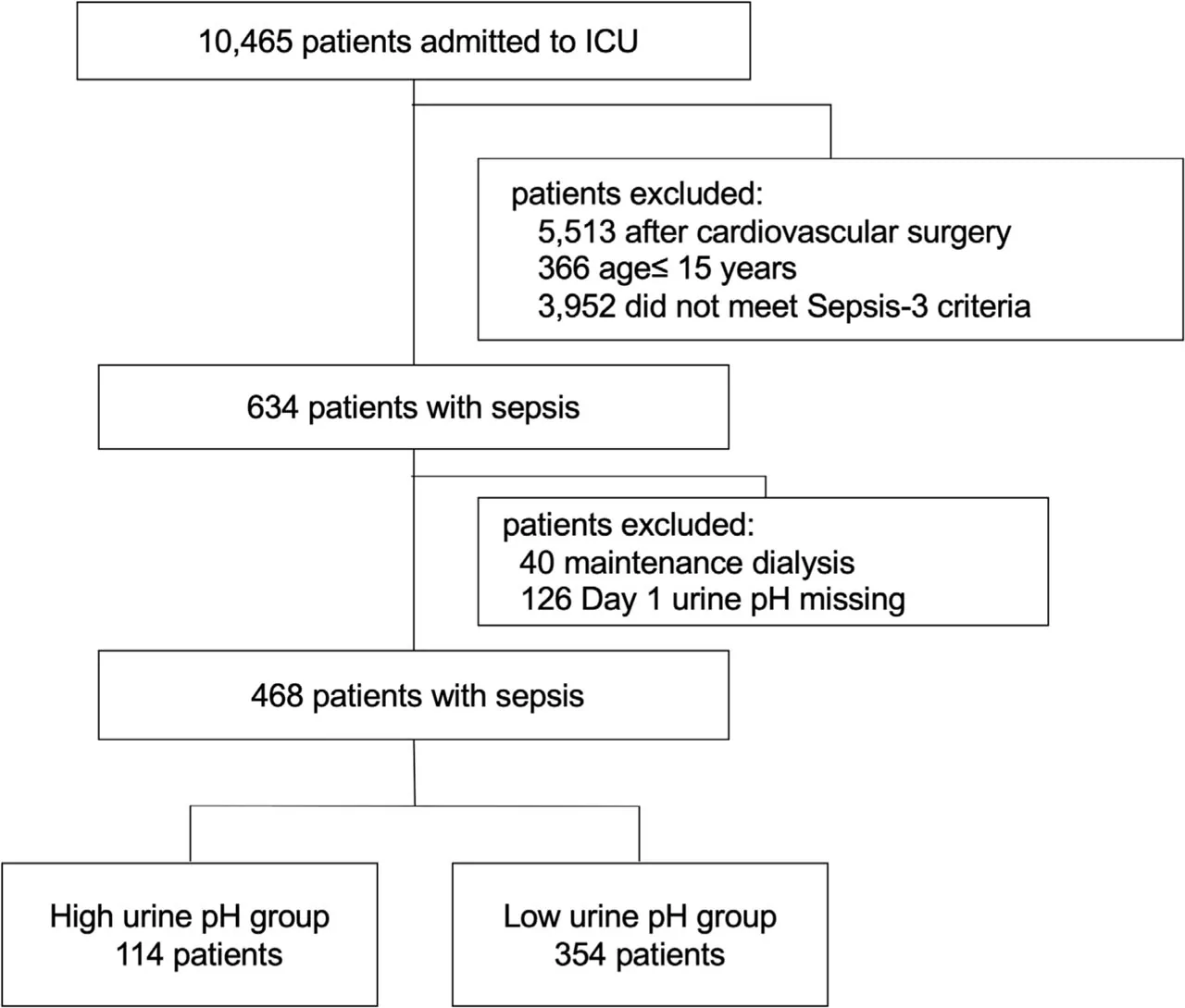
Patient selection flow diagram Patients were classified into high and low urine pH groups using a cutoff of day-1 urine pH 6.0 (high, >6.0; low, ≤6.0). ICU, intensive care unit

Patient characteristics for the 468 included patients are summarized in Table 1. The median age was 72.0 years (interquartile range [IQR], 62.0–78.0), and 61.8% (289/468) were male. The median BMI was 22.0 kg/m^2^ (IQR, 19.3–24.7). The median SOFA score on ICU admission was 10.0 (IQR, 7.0–12.0), and septic shock was present in 45.7% (214/468) of patients. The overall 90-day mortality rate was 25.6% (120/468). The median urine pH on the first day of ICU admission was 6.0 (IQR, 6.0–6.0). Missing values were observed only for D-dimer measurements (17 patients; 9 in the high urine pH group and 8 in the low urine pH group).

**Table 1:** Baseline characteristics of the overall cohort and according to urine pH on ICU day 1

	**Total**	**High urine pH group**	**Low urine pH group**	**P**
Number of patients	468	**114**	**354**	
Age (years)	72.0 (62.0–78.0)	70.0 (59.8–78.0)	73.0 (63.0–79.0)	0.122
Sex, male, n (%)	289 (61.8)	68 (59.6)	221 (62.4)	0.595
BMI (kg/m^2^)	22.0 (19.3–24.7)	21.2 (18.7–23.8)	22.2 (19.7–24.8)	0.085
SOFA score	10.0 (7.0–12.0)	9.0 (6.8–12.0)	10.0 (8.0–12.0)	0.028*
Respiration	2.0 (1.0–3.0)	2.0 (1.0–3.0)	2.0 (1.0–3.0)	0.099
Cardiovascular	3.0 (2.0–4.0)	3.0 (1.0–4.0)	3.0 (3.0–4.0)	0.111
Coagulation	0.0 (0.0–2.0)	1.0 (1.0–2.0)	0.0 (0.0–2.0)	0.506
Liver	0.0 (0.0–1.0)	0.0 (0.0–1.0)	0.0 (0.0–1.0)	0.372
Renal	0.5 (0.0–1.0)	0.0 (0.0–2.0)	1.0 (0.0–2.0)	0.038*
CNS	3.0 (1.0–4.0)	0.0 (0.0–2.0)	1.0 (0.0–2.0)	0.108
Charlson comorbidity index	6.0 (4.0–7.0)	5.0 (3.0–7.0)	6.0 (4.0–8.0)	0.243
WBC (×10^3^/μL)	9.70 (5.80–16.30)	10.3 (5.9–16.9)	9.7 (5.8–16.1)	0.452
Hemoglobin (g/dL)	10.05 (8.9–11.6)	10.2 (9.1–12.0)	10.0 (8.8–11.4)	0.205
Platelet count (×10^3^/μL)	171.5 (93.0–239.5)	173.50 (85.50–238.50)	170.50 (93.00–241.25)	0.989
TP (g/dL)	5.6 (4.9–6.4)	5.5 (5.0–6.3)	5.7 (4.8–6.4)	0.869
ALB (g/dL)	2.6 (2.1–3.0)	2.6 (2.1–3.0)	2.5 (2.1–3.0)	0.688
Tbil (mg/dL)	0.88 (0.54–1.30)	0.90 (0.52–1.21)	0.87 (0.55–1.33)	0.719
CRE (mg/dL)	1.07 (0.7–2.14)	0.92 (0.64–1.85)	1.11 (0.72–2.23)	0.078
CRP (mg/dL)	13.15 (5.34–21.39)	13.15 (4.80–22.69)	13.15 (5.37–21.23)	0.953
D-dimer (μg/mL)	6.4 (3.2–15.7)	6.7 (3.5–17.7)	6.4 (3.1–15.3)	0.45
pH	7.378 (7.306–7.430)	7.403 (7.335–7.458)	7.369 (7.300–7.420)	<0.001
pO_2_ (mmHg)	132.2 (86.4–243.3)	137.9 (94.4–230.8)	129.5 (84.8–245.1)	0.277
pCO_2_ (mmHg)	37.95 (31.1–44.23)	36.6 (29.7–44.0)	38.2 (31.7–44.4)	0.268
HCO_3_^−^ (mmol/L)	21.7 (18.725–24.375)	21.9 (19.5–25.0)	21.6 (18.4–24.3)	0.15
Base Excess (mmol/L)	−2.8 (−6.2–0.025)	−1.9 (−4.5–1.2)	−2.9 (−6.5–0.5)	0.015*
Lactate (mg/dL)	19.0 (11.0–34.25)	14.0 (10.0–30.0)	21.0 (12.0–37.0)	0.022*
Septic Shock, n (%)	214 (45.7)	40 (35.1)	174 (49.2)	0.009*
CRRT within 24 h, n (%)	210 (45.2)	45 (40.2)	165 (46.7)	0.224
Infection site, n (%)				0.199
Abdominal	190 (40.6)	42 (36.8)	148 (41.8)	
Respiratory	137 (29.3)	34 (29.8)	103 (29.1)	
Skin and soft tissue	41 (8.8)	13 (11.4)	28 (7.9)	
Urinary tract	29 (6.2)	12 (10.5)	17 (4.8)	
Blood	19 (4.1)	2 (1.8)	17 (4.8)	
Others	43 (9.2)	9 (7.9)	34 (9.6)	
unknown	9 (1.9)	2 (1.8)	7 (2.0)	

Data are presented as the median (interquartile range) or number (%), as appropriate. P values compare the High and Low urine pH groups. Continuous variables were compared using the Wilcoxon rank-sum test, and categorical variables using the chi-square test or Fisher's exact test, as appropriate.

An asterisk (*) indicates P < 0.05.

High urine pH group: urine pH > 6.0 on ICU day 1; Low urine pH group: urine pH ≤ 6.0 on ICU day 1.

For infection site, percentages are calculated within each urine pH group.

D-dimer values were missing in 17 patients (high urine pH group, n=9; low urine pH group, n=8).

ICU, intensive care unit; BMI, body mass index; SOFA, Sequential Organ Failure Assessment; CNS, central nervous system; WBC, white blood cell count; TP, total protein; ALB, albumin; Tbil, total bilirubin; CRE, creatinine; CRP, C-reactive protein; CRRT, continuous renal replacement therapy; pH, potential of hydrogen; PO_2_, partial pressure of oxygen; PCO_2_, partial pressure of carbon dioxide; HCO_3_^−^, bicarbonate.

### Primary Outcome: Association Between Day-1 Urine pH and 90-Day Mortality (Continuous Analysis)

Urine pH was analyzed as a continuous variable using a restricted cubic spline model (reference: pH 6.5) adjusted for covariates, including age, sex, BMI, SOFA score (excluding the CNS component), serum lactate level, and the presence of septic shock (Supplemental Figure 1). The spline curve demonstrated that higher urine pH values were associated with a lower adjusted hazard ratio for 90-day mortality.

The likelihood ratio test comparing the spline model (df = 4) with a linear model showed no significant evidence of nonlinearity (P = 0.331). Therefore, urine pH was treated as a continuous linear variable in the multivariable Cox regression model. In this model, each 0.5-unit increase in urine pH on Day 1 was significantly associated with a decreased risk of 90-day mortality (adjusted hazard ratio [HR]: 0.572; 95% confidence interval [CI]: 0.422–0.776; P < 0.001) (Table 2).

**Table 2:** Univariable and multivariable Cox proportional hazards regression analyses for 90-day mortality

	**Unadjusted**		**Adjusted**	
	**HR (95% CI)**	**P value**	**HR (95% CI)**	**P value**
Age	1.016 (1.003–1.030)	0.02*	1.009 (0.994–1.023)	0.244
Sex	1.349 (0.918–1.982)	0.127	1.103 (0.743–1.638)	0.626
BMI	0.938 (0.897–0.981)	0.005*	0.928 (0.886–0.972)	0.002*
SOFA score (excluding neurologic component)	1.015 (1.009–1.020)	<.001*	1.186 (1.113–1.264)	<.001*
Serum lactate (per 1 mg/dL)	1.012 (1.008–1.016)	<.001*	1.009 (1.004–1.014)	0.001*
Septic shock	2.452 (1.688–3.561)	<.001*	1.118 (0.712–1.756)	0.628
Urine pH (per 0.5-unit increase)	0.611 (0.457–0.816)	<.001*	0.572 (0.422–0.776)	<.001*

HR, hazard ratio; CI, confidence interval; BMI, body mass index; SOFA, Sequential Organ Failure Assessment.

The multivariate model included age, sex, body mass index, SOFA score (excluding the neurologic component), serum lactate level, septic shock, and Day1 urine pH.

* P < 0.05.

### Clinical Interpretation: Group Comparisons by Day-1 Urine pH – Baseline Characteristics

Urine pH was measured in 0.5-unit increments using a dipstick method, and the distribution was heavily concentrated at pH 6.0 on Day 1 (median [IQR]: 6.0 [6.0–6.0]). For clinical interpretability, the patients were stratified into two groups: Low urine pH group (pH ≤ 6.0) and High urine pH group (pH > 6.0).

Baseline characteristics and laboratory findings at ICU admission are shown in Table 1.

There were no significant differences between groups in terms of age, sex, or site of infection. The Low urine pH group exhibited significantly higher SOFA scores (10.0 vs. 9.0, P = 0.028), lower arterial blood pH and base excess, and higher serum lactate levels (all P < 0.05). In addition, the proportion of patients with septic shock was significantly higher in the Low urine pH group (49.2% vs. 35.1%, P = 0.009).

### Clinical Interpretation: Group Comparisons by Day-1 Urine pH – Kaplan–Meier and Cox Analysis

The 90-day mortality rate was 30.2% (107/354) in the Low urine pH group (pH ≤ 6.0) and 11.4% (13/114) in the High urine pH group (pH > 6.0). Kaplan–Meier survival analysis demonstrated significantly lower 90-day survival in the Low urine pH group than in the High urine pH group (log-rank p < 0.001) (Figure 2).

**Fig. 2.**
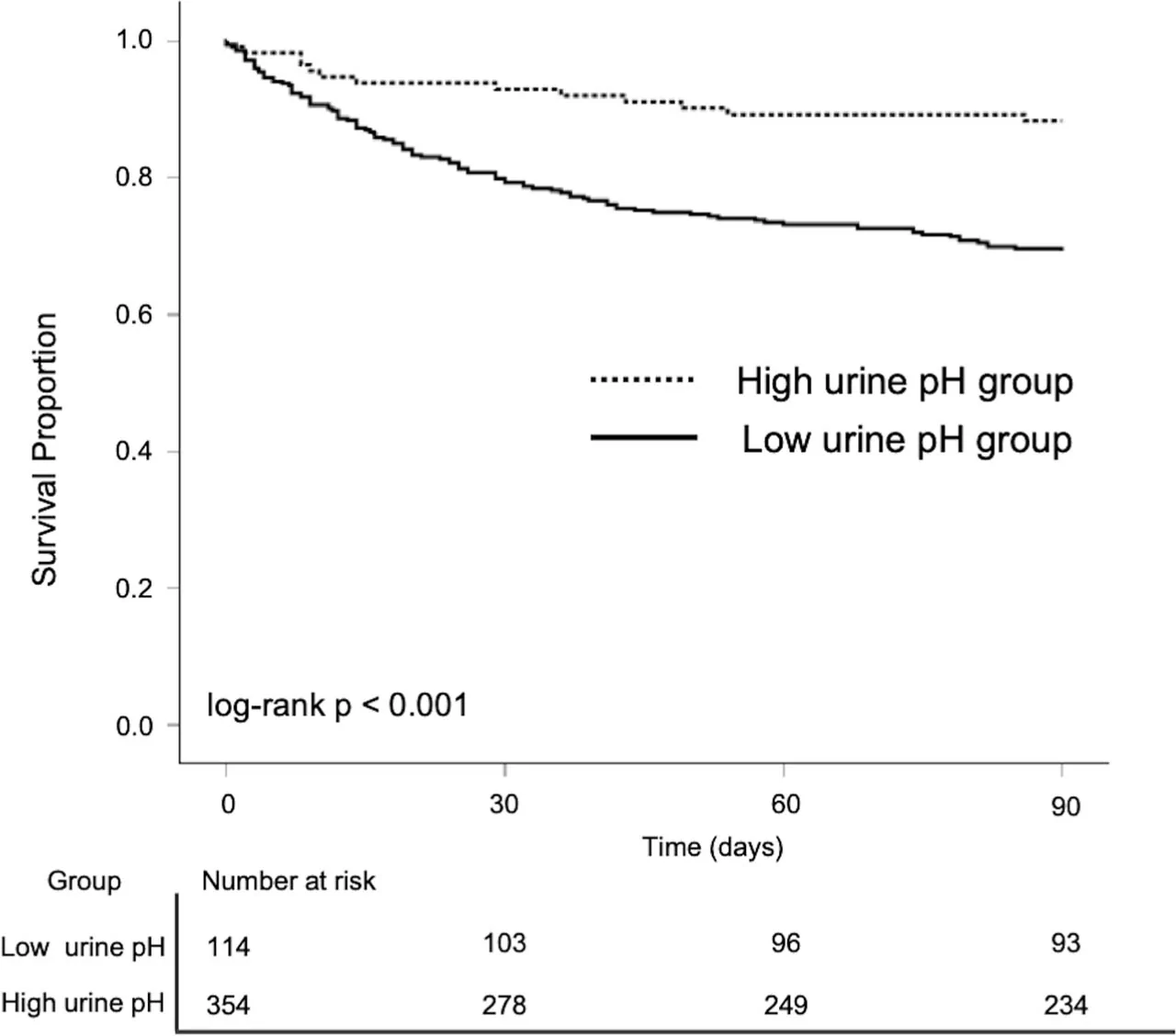
Kaplan–Meier survival curves by urine pH group Kaplan–Meier curves for 90-day survival in the high and low urine pH groups. Groups were defined using a Day 1 urine pH cutoff of 6.0 (high, >6.0; low, ≤6.0). P value were calculated using the log-rank test. Numbers at risk are shown below the plot.

In the multivariable Cox proportional hazards model adjusted for age, sex, BMI, SOFA score (excluding the CNS component), serum lactate level, and the presence of septic shock, the High urine pH group had a significantly lower risk of 90-day mortality than the low urine pH group (adjusted HR: 0.279; 95% CI: 0.147–0.529; P < 0.001) (Table 3).

**Table 3:** Univariable and multivariable Cox proportional hazards analyses for 90-day mortality using categorical urine pH

	**Unadjusted**		**Adjusted**	
	**HR (95% CI)**	**P value**	**HR (95% CI)**	**P value**
Age	1.016 (1.003–1.030)	0.02*	1.008 (0.994–1.023)	0.26
Sex	1.349 (0.918–1.982)	0.127	1.111 (0.749–1.649)	0.601
BMI	0.938 (0.897–0.981)		0.932 (0.891–0.974)	0.002*
SOFA score (excluding neurologic component)	1.015 (1.009–1.020)		1.186 (1.112–1.264)	<0.001*
Serum lactate (per 1 mg/dL)	1.012 (1.008–1.016)		1.010 (1.004–1.016)	<0.001*
Septic shock	2.452 (1.688–3.561)		1.061 (0.673–1.674)	0.799
Urine pH (>6.0 vs ≤6.0)	0.342 (0.192–0.608)		0.279 (0.147–0.529)	<0.001*

HR, hazard ratio; CI, confidence interval; BMI, body mass index; SOFA, Sequential Organ Failure Assessment.

### Clinical Interpretation: Group Comparisons by Day-1 Urine pH – SOFA Score and Urine Output

Supplemental Figure 2 shows the trends in urine output and SOFA scores from Day 1 to Day 3 after ICU admission, stratified by urine pH group. Urine output was significantly lower in the Low urine pH group throughout Days 1 to 3. Similarly, the total SOFA scores remained significantly higher in the Low urine pH group during the same period. In the component-wise SOFA analysis (Supplemental Figure 2), the Low urine pH group showed significantly higher scores for the circulatory system on Days 2 and 3, and for the renal system from Day 1 to Day 3.

### Secondary/Exploratory: Time-Dependent Association (Days 1–3)

To evaluate the temporal association between urine pH and 90-day mortality, we constructed separate Cox proportional hazards models using urine pH values from Day 1, Day 2, and Day 3 as the main explanatory variables. Urine pH on Day 1 remained significantly associated with 90-day mortality after adjustment, whereas the associations weakened on Days 2 and 3 and was not statistically significant (Figure 3).

**Fig. 3.**
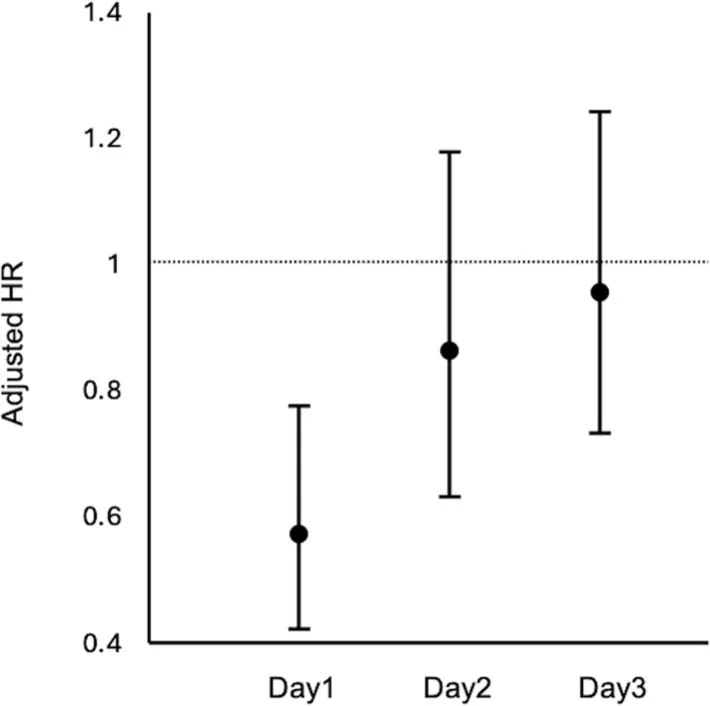
Association between urine pH and 90-day mortality across ICU Days 1–3 (adjusted hazard ratios) Adjusted hazard ratios (HRs) for 90-day mortality per increase in urine pH measured on ICU Day 1, Day 2, and Day 3 were estimated using Cox proportional hazards models. Points indicate adjusted HRs and vertical error bars indicate 95% confidence intervals. The dotted horizontal line denotes HR = 1.0 (no association). Models were adjusted for age, sex, body mass index, SOFA score (excluding the central nervous system component), serum lactate, and septic shock. HR, hazard ratio; CI, confidence interval; ICU, intensive care unit; SOFA, Sequential Organ Failure Assessment.

Adjusted hazard ratios (HRs) for 90-day mortality per increase in urine pH measured on ICU Day 1, Day 2, and Day 3 were estimated using Cox proportional hazards models. Points indicate adjusted HRs and vertical error bars indicate 95% confidence intervals. The dotted horizontal line denotes HR = 1.0 (no association). Models were adjusted for age, sex, body mass index, SOFA score (excluding the central nervous system component), serum lactate, and septic shock.

HR, hazard ratio; CI, confidence interval; ICU, intensive care unit; SOFA, Sequential Organ Failure Assessment.

### Exploratory: Change in Urine pH (Day 1–Day 3 Four Groups)

Among the 380 patients with available urine pH data on Days 1 and 3, we classified individuals into four groups based on the combination of urine pH values (cutoff = 6.0): Low–Low (n = 194), Low–High (n = 93), High–Low (n = 43), and High–High (n = 50). The baseline characteristics of each group are presented in Supplemental Table 2. This was an exploratory analysis limited to patients who survived until Day 3 and had available urine samples. No significant differences in the 90-day mortality were observed between the two subgroups within each stratum (Low vs. High) (Supplemental Figure 3).

Furthermore, changes in urine pH from Day 1 to Day 3 were not significantly associated with 90-day mortality risk Supplemental Table 3).

## Discussions

In this study, we evaluated the association between urine pH on the first day of ICU admission and the 90-day mortality in patients with sepsis. The principal finding was that lower urine pH on ICU Day 1 was independently associated with higher 90-day mortality, even after adjusting for age, disease severity, serum lactate levels, and septic shock. In contrast, urine pH on Days 2 and 3 and temporal changes in urine pH during the early ICU stay were not associated with 90-day mortality. A notable feature of this study was the finding that lower urine pH on the first day of ICU admission was associated with increased 90-day mortality. To our knowledge, few studies have specifically examined the association between urine pH and clinical outcomes in patients with sepsis, and this study addresses this gap.

A previous retrospective study in a general internal medicine ICU population reported that urine pH ≤5.5 was independently associated with ICU mortality [13]. However, several important differences should be considered when comparing that study with ours. The previous study included a heterogeneous ICU population and excluded patients with acute or chronic kidney failure, whereas our study focused exclusively on patients with sepsis, in whom acid–base disturbances and renal dysfunction are common manifestations of the disease process. In addition, the previous study dichotomized urine pH using a cutoff value of 5.5 and evaluated ICU mortality using logistic regression analysis, whereas we assessed urine pH as a continuous variable and examined its association with 90-day mortality using Cox proportional hazards models adjusted for established markers of sepsis severity. Despite these differences in population, definitions, and analytical approaches, both studies consistently suggest that lower urine pH is associated with adverse clinical outcomes.

In our cohort, the Low day-1 urine pH group had significantly higher SOFA scores at admission (particularly the renal component), along with elevated serum lactate levels, suggesting a population with more advanced multi-organ dysfunction, metabolic stress, and renal impairment. Given that these pathophysiological conditions are known to be associated with increased mortality in critically ill patients [8,15,16], the higher 90-day mortality observed in the Low day-1 urine pH group in our study appears clinically plausible. Importantly, even after adjusting for these between-group differences in multivariate analysis, lower urine pH remained independently and significantly associated with 90-day mortality. This finding suggests that urine pH may reflect underlying pathophysiological factors that are not fully captured by conventional severity indices. Urine pH is determined by the balance between systemic acid production and the tubular ability of the kidney to acidify and excrete acid in urine [17, 18]. In sepsis, factors such as microcirculatory dysfunction, inflammation, and metabolic reprogramming may impair tubular function, including urinary acidification, ammonium production/excretion [8]. As a result, urine pH may serve as an integrated output reflecting both systemic acid load and renal acid excretion response, thereby providing prognostic information. Given that urine pH can be measured rapidly and inexpensively at the bedside using only a urine specimen, without requiring additional invasive tests, it may serve as a highly implementable adjunct indicator for early severity assessment and risk stratification in the ICU setting. However, the observed association between low urine pH and poor prognosis in this study supports a hypothetical mechanism but does not establish causality. Further investigations are warranted to clarify this relationship.

In the Low day-1 urine pH group, urine output during Days 1–3 after ICU admission was significantly lower, and total SOFA scores were higher. Organ-specific SOFA scores also differed significantly, with higher renal scores from days 1 to 3 and higher circulatory scores on Days 2 and 3. These findings suggest that a lower urine pH on the first day of ICU admission may be associated with the severity of organ dysfunction over the subsequent days. The combination of lower urine output and higher renal SOFA scores in the low pH group may indicate a greater burden of sepsis-associated AKI, potentially driven by circulatory failure, systemic inflammation, and microvascular dysfunction. Although AKI is diagnosed based on serum creatinine levels and urine output [19,20,21], both measures are difficult to interpret accurately at the time of ICU admission because of potential confounders, such as fluid accumulation and variations in muscle mass [9, 22]. Taken together, urine pH upon ICU admission may serve as an integrated indicator of renal stress, reflecting systemic acid load, tubular function, hemodynamics, and the influence of therapeutic interventions, thereby providing additional information for early risk stratification. However, these findings do not imply that urine pH is a direct determinant of clinical outcomes. Instead, it may reflect the extent of organ dysfunction and renal stress during the acute phase of sepsis. Given that both daily SOFA scores and urine output can be affected by therapeutic interventions and missing data, caution is warranted when interpreting these associations.

In the exploratory analyses in this study, only urine pH on the first day of ICU admission was associated with 90-day mortality. Urine pH on Days 2 and 3 showed no significant association, nor did the change in urine pH from Day 1 to Day 3. During the acute phase of sepsis, therapeutic interventions, such as fluid resuscitation, diuretics, sodium bicarbonate administration, and renal replacement therapy, are often intensively administered within a short period. As a result, urine pH may fluctuate substantially [23], and its absolute values or temporal changes may not purely reflect illness severity or recovery but rather a mixture of endogenous and treatment-related influences. Therefore, although urine pH may be useful for early risk stratification at sepsis onset, caution is warranted when interpreting serial urine pH values as prognostic indicators.

However, the clinical utility of the urine pH as a therapeutic target remains unclear. A randomized clinical trial of urine alkalization using sodium bicarbonate showed that although urine pH increased in the intervention group, there was no significant difference in the incidence of AKI or clinical outcomes [24]. Similarly, observational studies involving critically ill patients, including those with sepsis, have suggested that urine pH may not be associated with AKI progression [8]. These findings support the notion that simple changes in urine pH may not directly translate into organ protection or improved prognosis and complement the results of the current study. Notably, the analysis of urine pH changes was limited to patients who survived until Day 3 and had available urine samples; therefore, these findings should be interpreted as exploratory.

A major strength of this study is that urine pH on the first day of ICU admission, a simple and readily obtainable parameter, was independently associated with 90-day mortality in a relatively large cohort of patients with sepsis. This association was observed independently of established severity indices. Future studies should aim to validate the external generalizability of these findings through multicenter prospective studies and explore more direct markers of renal acid excretion, such as urinary ammonium.

### Limitations

This study has certain limitations. First, this was a single-center, retrospective, observational study conducted at a tertiary academic medical center that frequently admits critically ill patients. Therefore, selection bias related to institutional characteristics, including patient demographics, treatment strategies, and testing protocols, may limit the generalizability of the findings. Although we adjusted for known confounders using multivariate analysis, residual confounding due to unmeasured variables (e.g., timing and dosing of fluid resuscitation, diuretics, sodium bicarbonate administration, renal replacement therapy, source control, and pre-existing renal dysfunction) cannot be excluded. Second, most participants were of Asian descent, and generalizability to other populations with different genetic backgrounds, dietary habits, or lifestyles remains unclear. Third, the analysis was limited to patients for whom urine pH measurements were available. While we excluded some cases because of missing values for primary exposure, no significant differences in baseline characteristics were observed between the included and excluded patients, suggesting that major selection bias was unlikely. However, the effect of unmeasured factors cannot be completely ruled out. Moreover, the analyses involving Day 2–3 urine pH and its temporal changes were restricted to patients who survived until Day 3 and had available urine samples. This may have led to an underestimation of the associations with outcomes owing to the exclusion of early deaths or patients with anuria/oliguria. Fourth, the urine pH was assessed using the dipstick method, which is commonly used in clinical practice. This method is subject to observer variability and limited resolution (0.5-unit increments), which introduces potential measurement error. Fifth, although urine pH and other laboratory tests were performed on the same day, the exact measurement times were not always synchronized, and not all samples were obtained immediately after ICU admission. Consequently, the results may reflect post-intervention effects, limiting interpretation as a snapshot of baseline status. Sixth, daily SOFA scores and urine output data are susceptible to the effects of clinical interventions and missing values and may be skewed toward survivors. Therefore, a causal interpretation of the association between low urine pH and progression of organ dysfunction must be approached with caution. Finally, urine output–based criteria were used to assess the renal SOFA score. However, urine output may be influenced by therapeutic interventions, such as diuretics, fluid loading, or renal replacement therapy, and may not fully reflect intrinsic renal dysfunction. Although serum creatinine–based evaluation has limitations, especially in patients receiving CRRT, future prospective studies accounting for therapeutic interventions are needed to validate these findings.

## Conclusion

In patients with sepsis admitted to the intensive care unit, a lower urine pH on the first day of ICU admission was independently associated with increased 90-day mortality. Urine pH is a simple and readily measurable parameter, and our findings suggest that it may serve as a complementary indicator for early risk stratification in the ICU setting.
